# Potential Biotechnological Strategies for the Cleanup of Heavy Metals and Metalloids

**DOI:** 10.3389/fpls.2016.00303

**Published:** 2016-03-15

**Authors:** Kareem A. Mosa, Ismail Saadoun, Kundan Kumar, Mohamed Helmy, Om Parkash Dhankher

**Affiliations:** ^1^Department of Applied Biology, College of Sciences, University of SharjahSharjah, UAE; ^2^Department of Biotechnology, Faculty of Agriculture, Al-Azhar UniversityCairo, Egypt; ^3^Department of Biological Sciences, Birla Institute of Technology and Science Pilani, K. K. Birla Goa CampusGoa, India; ^4^The Donnelly Centre for Cellular and Biomedical Research, University of Toronto, TorontoON, Canada; ^5^Stockbridge School of Agriculture, University of MassachusettsAmherst, MA, USA

**Keywords:** bioremediation, phytoremediation, rhizoremediation, transgenic, hyperaccumulation

## Abstract

Global mechanization, urbanization, and various natural processes have led to the increased release of toxic compounds into the biosphere. These hazardous toxic pollutants include a variety of organic and inorganic compounds, which pose a serious threat to the ecosystem. The contamination of soil and water are the major environmental concerns in the present scenario. This leads to a greater need for remediation of contaminated soils and water with suitable approaches and mechanisms. The conventional remediation of contaminated sites commonly involves the physical removal of contaminants, and their disposition. Physical remediation strategies are expensive, non-specific and often make the soil unsuitable for agriculture and other uses by disturbing the microenvironment. Owing to these concerns, there has been increased interest in eco-friendly and sustainable approaches such as bioremediation, phytoremediation and rhizoremediation for the cleanup of contaminated sites. This review lays particular emphasis on biotechnological approaches and strategies for heavy metal and metalloid containment removal from the environment, highlighting the advances and implications of bioremediation and phytoremediation as well as their utilization in cleaning-up toxic pollutants from contaminated environments.

## Introduction

Bioremediation is the use of natural and recombinant microorganisms for the cleanup of environmental toxic pollutants. It is considered a cost-effective and environmentally friendly approach. It relies on improved detoxification and degradation of toxic pollutants either through intracellular accumulation or via enzymatic transformation to lesser or completely non-toxic compounds (Brar et al., 2006). Many naturally or genetically modified microorganisms possess the ability to degrade, transform, or chelate various toxic chemicals and hence provide better strategies to combat environmental pollution. On a regular basis, scientists deploy either natural or modified microbes to remove contaminants, viz., heavy metals, metalloids, radioactive waste, and oil products from polluted sites (Dixit et al., 2015).

Plants possess the necessary genetic, biochemical, and physiological characteristics to establish themselves as the ultimate choice for soil and water pollutant remediation. Phytoremediation refers to a diverse collection of plant-based technologies that use either naturally occurring or genetically engineered plants to clean contaminated environments (Salt et al., 1995, 1998; Flathman and Lanza, 2010). Phytoremediation is a cost effective, green-clean technology with long-term applicability for the cleaning up of contaminated sites. However, the required time frame to clean-up contaminants from soil prevents its use on an industrial scale. It involves the cleaning up of contaminated soil and water by either root colonizing microbes or by the plants themselves and is best applied at sites with shallow contamination of organic and inorganic pollutants (Pilon-Smits, 2005). Due to this shortcoming, the utilization of biotechnological approaches involving high biomass fast growing crops for remediation purposes combined with biofuel production has gained momentum in recent years (Oh et al., 2013; Pidlisnyuk et al., 2014).

The development of new genetic tools and a better understanding of microbe and plant gene structures and functions have accelerated advancements in pathway-engineering techniques (referred to as designer microbes and plants) for improved hazardous waste removal. This review focuses on the accomplishments of biotechnological applications and strategies for environmental protection, detoxification, and the removal of heavy metals and metalloids. The current review article also examines recent developments and future prospects for the bio/phytoremediation of toxic pollutants from contaminated soil and water.

## Potential Strategies for Bioremediation

**FIGURE 1:**
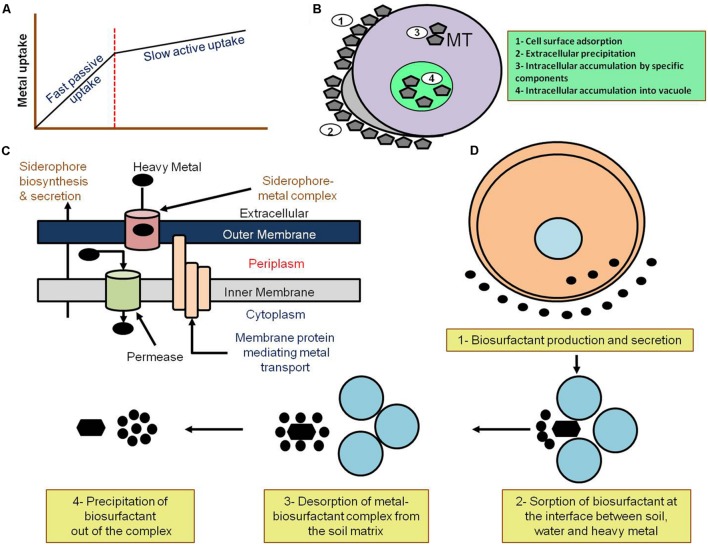
**Mechanism of microbial remediation. (A)** Passive and active heavy metal uptake by biological materials. The uptake of heavy metals can be either passive (fast) through adsorption onto the cell surface or any extracellular components such as polysaccharides, or alternatively active (slow) through sequestration of the heavy metals via interaction with metallothioneins (MT) into the cell. Adapted from Scragg (2005). **(B)** Mechanisms of heavy metal biosorption by bacterial cells. Bacterial biosorption of heavy metals through (1) cell surface adsorption, (2) extracellular precipitation, (3) intracellular accumulation through special components, such as metallothioneins (MT) or, (4) intracellular accumulation into vacuoles. Adapted from Banik et al. (2014). **(C)** Heavy metal remediation via siderophore formation. Bacterial heavy metal remediation takes place through formation of the siderophore aided by membrane protein-mediated metal transport and the formation of siderophore-metal complexes. Adapted from Banik et al. (2014). **(D)** Mechanism of bacterial heavy metal remediation through biosurfactant production. The precipitation of heavy metals takes place through sorption and desorption at the soil–water-heavy metal matrix leading to heavy metal precipitation. Adapted from Banik et al. (2014).

Microorganisms are mainly used in bioremediation to eliminate heavy metals (elements with densities above 5 g/cm^3^) from the polluted environment (Banik et al., 2014). In addition to the natural occurrence of heavy metals (Cobbina et al., 2015), they are widely used in industry, agriculture, and military operations. These processes have led to the continuous accumulation of heavy metals in the environment, which raises threats to public health and ecosystems. The high concentrations of heavy metals in the environment were also attributed to several life-threatening diseases, including cancer and cardiovascular ailments (Houston, 2007; Matés et al., 2010). The elimination of heavy metals requires their concentration and containment as they cannot be degraded by any biological, physical, or chemical processes (Naz et al., 2015). Therefore, employing microorganisms in heavy metal elimination and environmental cleaning is an effective approach due to their varied ability of interacting with heavy metals. For instance, microorganisms can transform heavy metals from one oxidative state or organic complex to another (Xiong et al., 2010). Mainly, microorganism-based remediation depends on the resistance of the utilized microbe to the heavy metal that is either activated independently or through metal stress (Naz et al., 2015). Microorganisms perform the remediation of heavy metals through three different processes (**Figure 1**):

### Biosorption and Bioaccumulation

Biosorption and bioaccumulation are processes by which the microorganisms, or biomass, bind to and concentrate heavy metals and contaminants from the environment (Joutey et al., 2015). However, both biosorption and bioaccumulation work in distinct ways. During biosorption, contaminants are adsorbed onto the sorbent’s cellular surface in amounts that depend on the composition and kinetic equilibrium of the cellular surface. Thus, it is a passive metabolic process (**Figure 1A**) that does not require energy/respiration (Velásquez and Dussan, 2009). Bioaccumulation, on the other hand, is an active metabolic process that needs energy and requires respiration (Vijayaraghavan and Yun, 2008; Velásquez and Dussan, 2009). Since contaminants (such as heavy metals) bind to the cellular surface of microorganisms during biosorption, it is a revisable process. In contrast, bioaccumulation is only partially reversible. Biosorption was also shown to be faster and to produce a greater number of concentrations (Velásquez and Dussan, 2009).

#### Biosorption

Biosorption is an emerging method that came into practice about two decades ago. It holds outstanding potential as a cost-efficient method for environmental cleaning and reducing heavy metal pollution resulting from industrial and agricultural sources (Fomina and Gadd, 2014; Javanbakht et al., 2014). This method depends on the sequestration of toxic heavy metals by the moieties of biosorbent cell surfaces (**Figure 1B**) such as those found in fungi/yeast, bacteria, and algae (Nilanjana et al., 2008). Applications of biosorption in bioremediation include heavy metal elimination from soil, landfill leachates and water as well as several other roles (Fomina and Gadd, 2014; Tran et al., 2015).

Several living organisms have been tested as potential biosorbents. This includes bacteria such as *Bacillus subtilis* and *Magnetospirillum gryphiswaldense*, fungi such as *Rhizopus arrhizus*, yeast such as *Saccharomyces cerevisiae* and algae such as *Chaetomorpha linum* and marine microalgae (seaweed) (Romera et al., 2006; Vijayaraghavan and Yun, 2008; Wang and Chen, 2008; Zhou et al., 2012). Furthermore, biomasses were proposed and investigated as a potential inexpensive and economical means of treating eﬄuents charged with toxic heavy metals. Biomasses such as industrial wastes (waste biomass of *Saccharomyces cerevisiae* from fermentation and the food industry), agricultural wastes (corn core) and other polysaccharide materials, were investigated and reviewed (Vijayaraghavan and Yun, 2008; Wang and Chen, 2008). Compared with other organisms, bacteria are considered outstanding biosorbents due to their high surface-to-volume ratios as well as several potential active chemosorption sites in their cell wall such as teichoic acid (Beveridge, 1989). Dead bacterial strains are also proposed as potential biosorbents with biosorption capacities that outperform living cells of the same strains. The biosorption capacity of chromium ions in the dead *Bacillus sphaericus* was increased by 13–20% in comparison with living cells of the same strain (Velásquez and Dussan, 2009).

The introduction of genetic engineering of bacteria and other microorganisms opened up new horizons for designing tailored organisms with engineered metal-binding peptides that possess improved affinity and selectivity for target metals to be used as heavy metal biosorbents. In bacteria, the surface display systems in various species such as *Staphylococcus xylosus* and *S. carnosus* were shown to express two different polyhistidyl peptides, i.e., His_3_-Glu-His_3_ and His_6_. The encoded gene products of the chimeric surface proteins were targeted to improve functionality in terms of metal binding as well as surface accessibility (Samuelson et al., 2000). Similarly, *Escherichia coli* and *Pseudomonas putida* showed improved phosphate biosorption through the immobilization of an intracellular phosphate-binding protein (PBP) onto its cell surface (Li et al., 2009). An early study demonstrated that surface-engineered gram-positive bacteria of two strains of *Staphylococci* resulted in the presentation of polyhistidyl peptides in a functional form (Samuelson et al., 2000). Another study on *E. coli* used genome engineering to express a Ni^2+^ transport system and overexpress pea metallothionein (MT) as a carboxyl-terminal fusion to glutathione S-transferase (GST-MT) simultaneously. The engineered *E. coli* cells demonstrated a promising ability in accumulating Ni^2+^ from diluted (<10 μM) solutions (Krishnaswamy and Wilson, 2000). A more recent study reported improved heavy metal biosorption capacities for *E. coli* cells engineered with mice MT1, demonstrating the potential of the genetic engineering approach in developing organisms with tailored and improved biosorption capacities (Almaguer-Cantú et al., 2011). The introduction of modern research technologies in genomics such as next generation sequencing (El-Metwally et al., 2014) and high throughput genome editing techniques (Bao et al., 2016) allowed the study of organisms with potential biosorption capacities that are expected to have several bioremediation applications in future.

#### Bioaccumulation

Bioaccumulation takes place when the absorption rate of the contaminant is higher than the rate of losing it. Thus, the contaminant remains contained and accumulated inside the organism (Chojnacka, 2010). Bioaccumulation is a toxicokinetic process that affects the sensitivity of living organisms to chemicals. Organisms can normally resist concentrations of chemicals up to certain levels, beyond which these chemicals become toxic and endanger the organism. The sensitivity of organisms to chemicals is highly variable depending on the types of organisms and chemicals involved (Mishra and Malik, 2013). Bioaccumulation candidate organisms should have a tolerance ranging between one or more contaminants to higher levels. Furthermore, they may demonstrate superior biotransformational capabilities, changing the toxic chemical to a non-toxic form which enables the organism to reduce the toxicity of the contaminant while keeping it contained (Ashauer et al., 2012; Mishra and Malik, 2013). Several different organisms are used for the study of bioaccumulation and as indicators for increased levels of pollutants, including plants (Somdee et al., 2015), fungi (Almeida et al., 2015), fish (Galal and Farahat, 2015; Ding et al., 2016), algae (Jaiswar et al., 2015), mussels (Diop et al., 2016), oysters (Mok et al., 2015), and bacteria (Diepens et al., 2015).

It was shown that bacteria produce metal-binding proteins such as metallothioneins (MTs) in order to increase the metal binding capacity as a response to increased metal exposure (**Figure 1B**) (Pazirandeh et al., 1998; Krishnaswamy and Wilson, 2000; Samuelson et al., 2000; Bae et al., 2001; Wernérus et al., 2001). In *E. coli*, for instance, the expression of metal binding peptides with the repetitive metal binding motif (Cys-Gly-Cys-Cys-Gly)_3_ as well as their high affinity and selectivity for target metals was investigated for potential use in bioremediation (Pazirandeh et al., 1998). In addition, recombinant *E. coli* strains that express a metal-binding peptide known as synthetic phytochelatins (PCs) with a repetitive metal-binding motif (Glu-Cys)_n_Gly were confirmed to have improved Cd^2+^ binding capability (Bae et al., 2001). Furthermore, *E. coli* strains with histidine-rich sequences fused to the LamB protein were shown to tolerate higher concentrations of Cd^2+^ and its counterpart in the equimolar mixture (Cu^2+^ or Zn^2+^; Kotrba et al., 1999).

In order to identify suitable bioaccumulation organisms, investigating the mechanisms and genes involved in the bioaccumulation process as well as the genes responsible for sensitivity/tolerance and the tolerated concentrations of different chemicals is crucial. Molecular biology techniques have been broadly used in such investigations (Mishra and Malik, 2013). The outstanding capacity of the GeoChip comprehensive microarray technique (He et al., 2007), which covers 424,000 genes in over 4,000 functional groups involved in several important biological processes, was used to investigate the bioaccumulation ability of several microbial groups to uranium (Van Nostrand et al., 2009). The DNA microarray technique was also used to identify genes regulated in response to exposure to high concentrations of heavy metals (Gorfer et al., 2009). Mass spectrometry-based proteomic techniques were employed to study the response to heavy metal stress at the translational level and changes in protein expression due to accumulation of high concentrations of toxic contaminants in cells (Italiano et al., 2009). Whole genome sequencing of microorganisms with potential bioaccumulation capacities helped in investigating candidate genes to be targeted for improving the bioaccumulation efficiency of the organism (Choi et al., 2015; Tan et al., 2015). Transcriptomics analysis has been used to delineate the roles of important genes involved in the bioaccumulation process and the difference in response to heavy metals between diverse organs in the same organism (Leung et al., 2014; Shi et al., 2015). Furthermore, bioinformatics and mathematical modeling has been utilized to investigate the properties and potentials of candidate organisms in order to predict the concentration of chemicals that can be tolerated by them (Stadnicka et al., 2012).

### Siderophore Formation

Siderophores are selective and specific iron chelating agents secreted by living organisms such as bacteria, yeasts, fungi (**Figure 1C**) and plants. Siderophores have a relatively low molecular weight and an extremely high binding affinity to trivalent metal ions (Fe^3+^), which is poorly soluble and predominantly found in oxygenated environments (Neilands, 1995; Chu et al., 2010). There are three different types of siderophores; namely, hydroxamate siderophores, catecholates (phenolates) siderophores and carboxylate siderophores. The hydroxamate siderophores are the most common group of siderophores. It consists of C (=O) N-(OH) R, where R is an amino acid and its derivative which are mainly produced by bacteria and fungi. The catecholate siderophores binds with iron through the formation of the hexadentate octahedral complex. Several well-known bacteria such as *E. coli* and *Salmonella typhimurium* produce these type of siderophores (Searle et al., 2015). The carboxylate siderophores bind to iron ions through the carboxyl and hydroxyl groups, and is produced by rhizosphere bacteria such as *Rhizobium* as well as several other types of bacteria. The types of siderophores are overviewed in Chu et al. (2010).

It was shown that trivalent forms of metals (other than iron) having similar chemistry can stimulate bacteria to produce siderophores, for instance, Al, Ga, and Cr (Schalk et al., 2011). Thus, the positive effects of siderophores on remediation such as reducing bioavailability and metal toxicity is not limited to iron but can be extended to several other toxic heavy metals. Stimulation of siderophore synthesis by heavy metals in the presence of high iron concentrations in *Pseudomonas aeruginosa* and *Alcaligenes eutrophus* bacteria was reported in an early study by Koedam et al. (1994). Furthermore, the reduction of copper toxicity by siderophore-mediated complexation in cyanobacteria was also confirmed (Stone and Timmer, 1975). Most recent reports have shown that several proteins involved in siderophore uptake have been revealed, including their detailed structure and sequences comprising of several outer membrane siderophore transporters and soluble periplasmic siderophore-binding proteins (Chu et al., 2010).

Systems biology approaches have been recently used in the investigation of siderophores formation on an unprecedented scale. Genomics and metagenomics were used to evaluate the environmental consequences of the devastating 2011 earthquake and tsunami in Japan that drastically altered the soil environment (Hiraoka et al., 2016). Mass spectrometry-based proteomic techniques also served a central role in the identification of siderophore-related proteins and the functional roles they play. The Proteomic Investigation of Secondary Metabolism (PrISM) platform was used to discover two new siderophores from the *Streptomyces* species, i.e., gobichelin A and B (Chen et al., 2013). The analysis of draft genome sequences and high-resolution proteomics data of *Streptomyces lilacinus* using the proteogenomics approach accelerated the identification of new putative threonine and the DHB-containing siderophore (Albright et al., 2014). Bioinformatics analysis, coupled with high throughput experimental techniques, demonstrated a remarkable approach in addressing challenges such as identifying the secondary metabolites produced by cryptic genes in bacteria (Park et al., 2013) as well as creating online resources for the field (Flissi et al., 2016). Systems biology is a very promising approach for the study of genes, mechanisms, and signaling pathways during siderophores formation.

### Biosurfactants Production

Surfactants or surface-active agents are substances that alter the prevailing conditions of surfaces through adsorption leading to lower surface tension between liquids or between a liquid and a solid (Ron and Rosenberg, 2001; Reznik et al., 2010). Thus, they can generally classified into (1) high molecular weight polymers binding tightly to solid surfaces and (2) low molecular weight molecules efficiently lowering the surface and interfacial tensions (Ron and Rosenberg, 2001). Biosurfactants are surfactants produced or secreted by living organisms such as microbes (**Figure 1D**). Although biosurfactants have been commonly used for organic pollutants remediation, several studies have also reported that biosurfactants are able to complex and remediate heavy metals such as Cd, Pb, and Zn (Maier and Soberón-Chávez, 2000; Mulligan, 2005).

Rhamnolipids are a major class of biosurfactants that are produced by *P. aeruginosa* and several other organisms (Maier and Soberón-Chávez, 2000). They are glycolipids with rhamnose moiety comprising of a glycosyl head group and a 3-(hydroxyalkanoyloxy) alkanoic acid (HAA) fatty acid as the tail (Ochsner et al., 1994; Desai and Banat, 1997). Rhamnolipids have several potential applications in industry and as additives for environmental remediation (Müller et al., 2012). The ability to capture heavy metal ions through electrostatic or complexation techniques has been attributed to anionic biosurfactants. These complexations lead to an increase in the apparent solubility of metals (Rufino et al., 2012). Therefore, the bioavailability of metals is affected through their reduction by common metabolic by-products, which leads to the formation of less soluble metal salts including phosphate and sulfide precipitates (Maier et al., 2000). There are several other biosurfactants produced by a wide range of bacterial and yeast species such as exocellular polymeric surfactants in the form of polysaccharides, proteins, lipopolysaccharides, lipoproteins or complex mixtures. For instance, several species of *Acinetobacter* demonstrated robust production of high molecular weight emulsifiers (Ron and Rosenberg, 2001).

Recently, the process of biosurfactant discovery benefited greatly from harnessing the power of modern high throughput and systems biology techniques. Metagenomic approaches were employed to discover novel biosurfactants that are useful for marine ecosystems (Jackson et al., 2015). The localization process of the biosurfactant family sophorolipids produced by *Starmerella bombicola* was investigated using mass spectrometry-based proteomics resulting in the discovery of localization regulators (Ciesielska et al., 2014). The rapidly accumulated knowledge on biosurfactants derived by computational biologists and bioinformaticians allowed the development of computational methods that are especially tailored for biosurfactant discovery as well as online resources to collect the accumulated data/knowledge in centralized resources. For instance, the BioSurf database provides curated information about biosurfactants and their organisms, proteins, metabolic pathways as well as associated algorithms and bioinformatics tools used for biosurfactant discovery (Oliveira et al., 2015). Biosurfactants and their applications have been reviewed in detail in several recent reports (Müller et al., 2012; Kiran et al., 2015; Mnif and Ghribi, 2015).

## Potential Strategies for Phytoremediation

**FIGURE 2:**
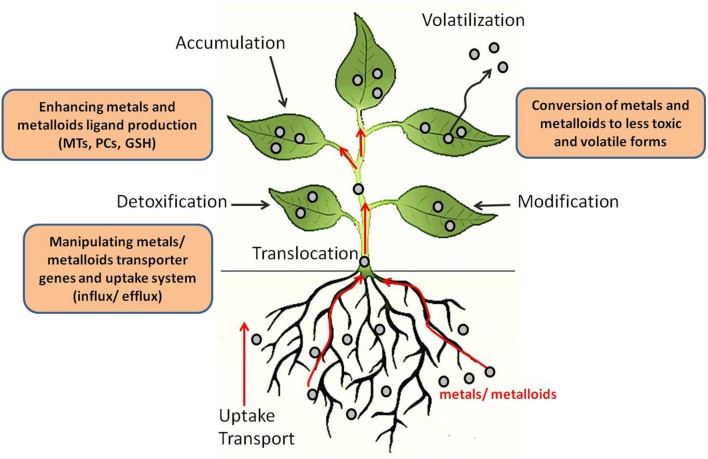
**Potential biotechnological strategies for phytoremediation.** Toxic elements can be mobilized and transported (influx) into roots through plasma membrane transporters. They can then be transported (eﬄux) out of the roots into the xylem and translocated into the shoots. At this stage, plant tolerance to toxic elements may be enhanced through manipulation of influx/eﬄux transporters or by increasing the levels of ligands/chelators. Volatilization of the toxic elements can be achieved through enzymes that modify these toxic elements. Chelators or eﬄux transporters can also be used to export the toxic elements out of the cytosol and into vacuoles or the cell wall. Adapted from Dhankher et al. (2011).

Plants have been used for phytoremediation of toxic metals and metalloids but this process has been slow and largely rendered ineffective due to phytotoxicity of heavy metals to plants (Dhankher et al., 2011). Natural hyperaccumulators of heavy metals are also available. However, they lack the critical biomass required for effective phytoremediation and are also restricted to particular geo-climatic conditions. Genetic engineering approaches to enhance plant tolerance and the accumulation of toxic metals holds great potential for phytoremediation. In addition, several recent studies employing omics technologies including genomics, transcriptomics, proteomics, and metabolomics have been carried out to elucidate the genetic determinants and pathways involved in heavy metal and metalloid tolerance in plants. Biotechnological approaches are currently being used for the phytoremediation of heavy metals and metalloids such as mercury (Hg), cadmium (Cd), lead (Pb), selenium (Se), copper (Cu), and arsenic (As). Three main biotechnological approaches are being used to engineer plants for phytoremediation of heavy metals and metalloids (**Figure 2**): (1) manipulating metal/metalloid transporter genes and uptake systems; (2) enhancing metal and metalloid ligand production; (3) conversion of metals and metalloids to less toxic and volatile forms (Kotrba et al., 2009).

### Manipulating Metal/Metalloid Transporter Genes and Uptake System

Improved metal tolerance and accumulation has been achieved in different plant species by manipulating metal transporters (**Figure 2**). For instance, *Arabidopsis thaliana* overexpressing yeast YCF1 (Yeast Cadmium Factor 1) resulted in enhanced tolerance to Pb(II) and Cd(II) and accumulated higher amounts of these metals in plants (Song et al., 2003). YCF1 is involved in Cd transport into vacuoles by conjugation with glutathione (GSH). Overexpression of full length *Nicotiana tabacum* plasma membrane channel protein (*NtCBP4*) showed Pb^2+^ hypersensitivity and enhanced Pb^2+^ accumulation in the transgenic plants. The overexpression of a truncated version of *NtCBP4* produced by deletion of its C-terminal, calmodulin-binding domain and part of the putative cyclic nucleotide-binding domain exhibited improved tolerance to Pb^2+^ and less accumulation of Pb^2+^ (Sunkar et al., 2000). Furthermore, T-DNA mutants of the *Arabidopsis* CNGC1 gene (cyclic nucleotide-gated ion channel 1), which encodes a homologous protein to *NtCBP4*, also conferred Pb^2+^ tolerance. These results suggest that NtCBP4 and AtCNGC1 play a role in the Pb^2+^ transport pathway (Sunkar et al., 2000; Zeng et al., 2015). Tobacco plants expressing *CAX2* (calcium exchanger 2) accumulated more Ca^2+^, Cd^2+^, and Mn^2+^ and were more tolerant to elevated Mn^2+^ levels. Expression of *CAX2* in tobacco increased Cd^2+^ and Mn^2+^ transport in isolated root tonoplast vesicles (Hirschi et al., 2000).

Arsenic (As) is a highly toxic metalloid which is classified as a group I carcinogen for humans by the International Agency for Research on Cancer (IARC) (IARC Monographs, 2004). Inorganic As forms, arsenate (AsV) and arsenite (AsIII), are common in the environment and more toxic than its organic forms (Dhankher et al., 2011). Arsenate is a phosphate analog taken up via phosphate uptake systems in plants (Catarecha et al., 2007; Zhao et al., 2009). Recent studies have shown that arsenite is transported in plants by members of the aquaporins family (Bienert et al., 2008; Zhao et al., 2009; Mosa et al., 2012). Strategies of developing transgenic plants for arsenic (As) phytoremediation include enhancing plant uptake for phytoextraction, decreasing plant uptake, improving the plants’ tolerance to As contamination, and increased methylation for enhanced food safety; these are reviewed in depth by Zhu and Rosen (2009) and Dhankher et al. (2011). Other metalloids like antimonite (SbIII), Silicon (Si) and Boron (B) have been reported to be transported through aquaporin channel proteins (Bienert et al., 2008; Mosa et al., 2011, 2016; Mosa, 2012; Kumar K et al., 2014).

Recently, genome-wide expression analysis of rice roots exposed to different heavy metals and metalloids; As(V), Cd, Cr(VI), and Pb, revealed several differentially expressed common as well as unique genes (Dubey et al., 2014). Interestingly, genes belonging to different transporter families such as major facilitator superfamily antiporter were identified (Dubey et al., 2014). Furthermore, Cu tolerant genes have been identified in the *Paeonia ostii* plant using the *de novo* transcriptome sequencing approach (Wang et al., 2016).

### Enhancing Metals and Metalloids Ligand Production

There are several reports of using Cys-rich peptides such as MTs, PCs, and GSH as metal-binding ligands for the detoxification or accumulation of heavy metals (**Figure 2**). For example, *A. thaliana* overexpressing a MT gene, PsMTA from pea (*Pisum sativum*), showed increased Cu^2+^ accumulation in roots (Evans et al., 1992). When the *E. coli gshII* gene encoding GSH synthetase (GS) was overexpressed in the cytosol of Indian mustard (*Brassica juncea*), transgenic plants demonstrated enhanced tolerance to and accumulated significantly more Cd than wild-type (WT) plants (Liang Zhu et al., 1999). Shrub tobacco overexpressing the wheat *TaPCS1* gene encoding PC synthase increased its tolerance to Pb and Cd significantly; transgenic seedlings grown in soil containing 1572 ppm Pb accumulated double the amount of Pb than WT plants (Gisbert et al., 2003). Arsenic (As) tolerance in plants can also be enhanced by modifying GSH and PCs. When two bacterial genes, *E. coli* arsenate reductase (*arsC*) and γ-glutamylcysteine synthetase (*γ-ECS*), were co-expressed in *Arabidopsis*, the double transgenic plants grown in the presence of 125 μM sodium arsenate accumulated threefold more As in the aboveground biomass and showed almost 17-fold higher biomass than WT plants (Dhankher et al., 2002). Constitutive overexpression of *AtPCS1* in *A. thaliana* showed enhanced tolerance to arsenate but failed to enhance As accumulation (Li et al., 2004). Similarly, overexpression of AtPCS1 in *B. juncea* showed a moderate increase in tolerance to arsenate, but not As accumulation in shoots (Gasic and Korban, 2007). These studies showed that manipulation of genes for increasing the production of metal chelation agents hold great potential for improving heavy metal and metalloid tolerance and accumulation in plants.

Recently, the *de novo* sequencing approach for radish (*Raphanus sativus L.*) root under Cd stress has been used to identify differentially expressed genes and microRNAs (miRNAs) involved in Cd-responsive regulatory pathways. Different candidate genes were suggested to play a major role on Cd accumulation and detoxification, particularly those encoding for MTs, PCs, and GSHs as well as other genes belonging to ABC transporters and zinc iron permease (ZIPs) (Xu et al., 2015). Similarly, RNA-Seq and *de novo* transcriptome analysis showed that 1561 unigenes were down-regulated and 1424 unigenes were up-regulated, respectively, in radish roots under chromium stress (Xie Y. et al., 2015). Here, several transcription factors such as Cr stress-responsive genes involved in signal transduction, chelate compounds and antioxidant biosynthesis have been proposed (Xie P. et al., 2015). Furthermore, the degradome sequencing approach has been used to identify miRNAs and their target genes under Pb stress in *Platanus acerifolia* tree plants (Wang et al., 2015).

### Conversion of Metals and Metalloids to Less Toxic and Volatile Forms

Several research groups have focused their efforts on developing phytoremediation strategies for Se and Hg using biotechnological approaches employing the conversion of these metals to less toxic and volatile forms (**Figure 2**). Selenium (Se) is an essential micronutrient for many organisms. However, in excess, it is very toxic and is a worldwide environmental pollutant (Zwolak and Zaporowska, 2012). Se occurs naturally in soil, and is chemically similar to sulfur (S). Therefore, plants uptake the inorganic and organic forms of Se via S transporters and metabolize them to volatile forms through S assimilation pathways to relatively non-toxic forms, such as dimethylselenide (DMSe). As reviewed by Pilon-Smits and LeDuc (2009), biotechnological strategies that have been used for selenium phytoremediation have focused on enhancing Se tolerance, accumulation, and volatilization. A constitutive overexpression of *A. thaliana* ATP sulfurylase (APS), converting selenate to selenite, in *Brassica juncea* showed enhanced reduction of selenate to organic Se forms in the APS overexpressed plants, whereas WT plants accumulated mainly selenate. The APS transgenic plants showed enhanced tolerance to selenate as compared to WT plants (Pilon-Smits et al., 1999). *Arabidopsis* plants expressing a mouse selenocysteine lyase (Scly) gene showed enhanced shoot Se concentrations (up to 1.5-fold) compared to the WT (Pilon et al., 2003). The overexpression of the SMT gene from the Se hyperaccumulator *Astragalus bisulcatus* in *A. thaliana* and *B. juncea* improved the tolerance of transgenic plants to selenium and enhanced Se accumulation in shoots. The transgenic plants also increased Se volatilization rates (LeDuc et al., 2004). Recently, Small RNA and degradome sequencing analyses were used to identify several miRNAs induced by Se treatment in the Se hyperaccumulator *Astragalus chyrsochlorus* plant callus (Cakir et al., 2016). Furthermore, differentially expressed genes in *A. chyrsochlorus* under selenate treatment were identified using *de novo* transcriptome analysis (Çakir et al., 2015).

Mercury (Hg) is a global pollutant threatening human and environmental health cycling between air, water, sediment, soil, and organisms (Moreno et al., 2005). The inorganic forms of mercury, elemental metallic Hg(0) or ionic Hg(II), are commonly liberated into the environment (Ruiz and Daniell, 2009). Strategies used for mercury phytoremediation have employed two bacterial genes; merA, encoding mercuric ion reductase, and merB, encoding organomercurial lyase, to convert mercury into less toxic forms (Meagher, 2000; Dhankher et al., 2011). Various plant species such as *A. thaliana* (Rugh et al., 1996), yellow poplar (Rugh et al., 1998), cottonwood (Che et al., 2003), rice (Heaton et al., 2003), and tobacco (Heaton et al., 2005) constitutively expressing modified *merA* were resistant to levels up to 25–250 μM HgCl_2_ and exhibited significant levels of Hg(0) volatilization as compared to control plants. Similarly, expression of a modified bacterial *merB* in *Arabidopsis* showed a significant resistance to high levels of monomethylmercuric chloride and phenylmercuric acetate relative to control plants (Bizily et al., 1999). *Arabidopsis* plants expressing both genes, *merA* and *merB*, grow on 50-fold higher methylmercury concentrations than WT plants and up to 10-fold higher concentrations than plants that express *merB* alone. Transgenic plants were also seen to detoxify organic mercury by converting it to volatile and significantly less toxic elemental mercury (Bizily et al., 2000).

## Rhizoremediation: The Combinatorial Effects Of Bio/Phytoremediation

Rhizoremediation is the combination of two approaches, i.e., phytoremediation and bioaugmentation, for cleaning contaminated substrates. Rhizoremediation refers to the exploitation of microbes present in the rhizosphere of plants utilized for phytoremediation purposes. Heavy metal resistant rhizospheric and endophytic bacteria are ecologically friendly and cost effective toward the reclamation of heavy metal polluted soil (Weyens et al., 2009; Rajkumar et al., 2010). The exploitation of metal resistant siderophore-producing bacteria (SPB) present near the rhizosphere provide nutrients (particularly iron) to the plants that could reduce the deleterious effects of metal contamination (Dimkpa et al., 2008; Sinha and Mukherjee, 2008). The siderophore not only solubilizes iron from minerals or organic compounds, it can also form stable complexes with environmental concern metals such as Al, Cd, Cu, Ga, In, Pb, Zn, and radionuclides (Neubauer et al., 2000; Rajkumar et al., 2010). Bacteria, mainly plant growth promoting rhizobacteria (PGPR), and fungi, mainly arbuscular mycorrhizal fungi (AMF), are used as pure cultures or co-cultures for bioaugmentation. PGPR such as *Agrobacterium, Alcaligenes, Arthrobacter, Azospirillum, Bacillus, Burkholderia, Serretia, Pseudomonas*, and *Rhizobium* are generally used for metal extraction with plants (Carlot et al., 2002; Glick, 2003). The siderophore synthesized by *P. fluorescens* improves Fe uptake in tomato, carnation, oats, vine, and maize (Dussa et al., 1986; Sharma and Johri, 2003). High levels of resistance to Cd (300 mg/L), Zn (730 mg/L), and Pb (1400 mg/L) were reported for a plant growth-promoting bacterial (PGPB) strain of *Bacillus* sp. (SC2b), which was isolated from the rhizosphere of *Sedum plumbizincicola* grown in Pb/Zn mine soils (Ma et al., 2015).

The siderophore-producing and arsenate-reducing *Pseudomonas* sp. bacterial strain plays a key role in the ability to convert arsenate to arsenite as well as promote plant growth and increase in the biomass of the fern *Pteris vittata* (Lampis et al., 2015). This suggests that the presence of rhizobacteria in soil can improve the efficiency of arsenic phytoextraction in hyperaccumulator plant species as well (Lampis et al., 2015). Abou-Shanab et al. (2006) reported that the bacterial strain *Microbacterium oxydans* AY509223 plays a role in nickel (Ni) mobilization and increased Ni uptake of *Alyssum murale* grown in low, medium, and high Ni soils by 36.1, 39.3, and 27.7%, respectively. The results provided potential development of inoculum for enhanced uptake during commercial phytoremediation or phytomining of Ni. The rhizo- and endophytic bacterial communities of *Prosopis juliflora* also harbor some novel heavy metal-resistant bacteria. Cr-resistant rhizobacteria showed resistance to Cr up to 3000 mg l^-1^ and provided tolerance against other toxic heavy metals such as Cd, Cu, Pb, Zn, and high concentrations of NaCl (Khan et al., 2014). The three different endophytic bacterial strains, viz., *Pantoea stewartii strain ASI11, Microbacterium arborescens strain HU33*, and *Enterobacter* sp. *strain HU38* improved plant growth and heavy metal removal from tannery eﬄuent contaminated soils, and showed that these bacteria play a role in improving the phytoremediation efficiency of heavy metal degraded soils (Khan et al., 2014).

Genomic analysis of three legume growth-promoting rhizobia (*Mesorhizobium amorphae* CCNWGS0123, *Sinorhizobium meliloti* CCNWSX0020 and *Agrobacterium tumefaciens* CCNWGS0286) that survive at regions with high levels of heavy metals in China confirmed the existence of different transporters involved in nickel, copper, zinc, and chromate resistance (Xie P. et al., 2015). In another report, Zhao et al. (2015) used an RNA-seq approach to investigate the genes associated with Cd stress in the Dark Septate Endophytic (DSE) fungal Cd-tolerant strain *Exophiala pisciphila* isolated from the roots of the Poaceae plant *Arundinella bengalensis.* The study showed that 228 unigenes involved in different pathways were associated with Cd-tolerance (Zhao et al., 2015). Recently, a novel metal transporter homology to natural resistance associated macrophage protein (Nramp) isolated from *Exophiala pisciphila* has been reported to increase Cd^2+^ sensitivity and accumulation when heterologously expressed in yeast (Wei et al., 2016). The combinatorial effects of bioaugmentation and phytoremediation leading to rhizoremediation may solve the problems encountered during the application of both techniques individually. Moreover, phytoextraction could be enhanced through the application of genetically engineered plant associated microorganisms.

## Conclusion and Future Aspects

Phytoremediation is an eco-friendly ‘green-clean’ technology that has tremendous potential to be utilized in the cleaning up of heavy metals and organic pollutants. For organic pollutants, plants, and rhizospheric bacteria have demonstrated the ability to detoxify and mineralize the former to harmless products that can be removed without causing accumulation. There are also a few reports of utilizing phytoremediation to successfully remove TCE and other organic compounds (Dhankher et al., 2011). However, in the case of toxic metals, plants can uptake, detoxify, translocate, and accumulate them in the aboveground biomass, which has to be then harvested for metal recovery. Despite tremendous potential for the application of phytoremediation in the cleaning up of contaminated soil, sediment, and water, it has not been commercialized and used extensively on a large scale.

There are many reports of heavy metal/metalloid uptake, detoxification, and accumulation but most of these were described at the laboratory scale in model plants (Dhankher et al., 2011; Hossain et al., 2012; Ovečka and Takáč, 2014). According to our knowledge, none of these studies have been applied in the field for heavy metal detoxification and phytoremediation thus far. Furthermore, progress toward commercializing the phytoremediation of heavy metals and metalloids has been hampered due to a lack of complete understanding of the metal uptake process from soil to roots, translocation from roots to shoots and accumulation in the biomass tissues. Several recent studies have attempted to unravel the mechanism of heavy metal and metalloid transport and accumulation in plants using transcriptomic and proteomics approaches (Cvjetko et al., 2014). Additionally, metabolomic analysis can help to identify the metabolites associated with heavy metal and metalloid stresses, which can be further mapped to its metabolic pathways to identify the related candidate genes (Kumar A et al., 2014). One intriguing approach to enhance our knowledge about heavy metal and metalloid metabolism in plants is to develop suitable techniques for imaging. Efforts have been made to employ Laser ablation inductively coupled plasma mass spectrometry (LA-ICP-MS), Matrix Assisted Laser Desorption Ionization (MALDI) and Fourier Transform Ion Cyclotron Resonance Mass Spectrometry (FT-ICR-MS) toward this aim (Jones et al., 2015). However, more efforts are needed to enable imaging visualization and determination of metal and metalloid localization and distribution in plant tissues. Despite recent progresses in biotechnological applications and the availability of complete genome sequences of several plants species, the potential of phytoremediation has still not been fully exploited for the successful application of this technology on a commercial scale for the cleaning of contaminated soil and water. Another major factor for the lack of progress in this area is inadequate funding for phytoremediation research.

**FIGURE 3:**
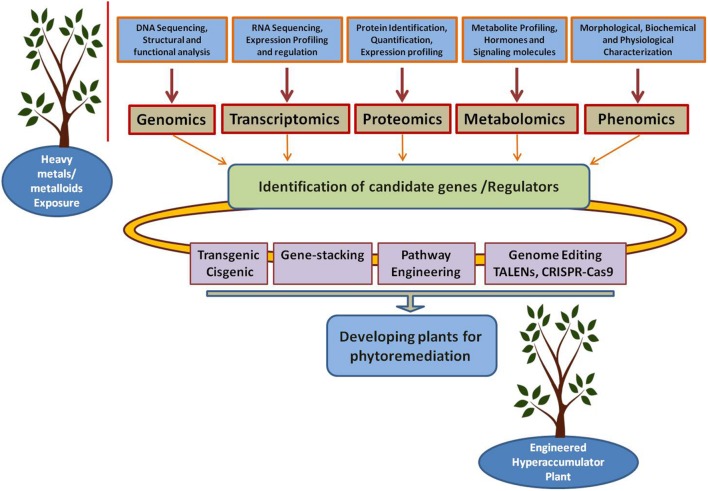
**Integration of “Omics” tools for developing plants for phytoremediation.** Genomics, transcriptomics, proteomics, metabolomics, and phenomics could help on identifying the candidate genes which can be used for developing plants for phytoremediation through different approaches including transgenic, cisgenic, gene- stacking, metabolic engineering, and genome editing.

Next generation sequencing was used to study the whole genomes and transcriptomes of several heavy metal-tolerant organisms (Hu et al., 2005; He et al., 2011; Peña-Montenegro and Dussán, 2013). Mass spectrometry-based proteomics is extensively used to study heavy metal and other forms of stresses in candidate organisms including plants (Hossain and Komatsu, 2012; Cvjetko et al., 2014), bacteria (Zakeri et al., 2012), and marine organisms (Muralidharan et al., 2012). Furthermore, proteogenomics, the alliance between proteomics and genomics (Helmy et al., 2012), is being used to study the genomic and proteomic properties of microorganisms that tolerate high concentrations of contaminants and high levels of stress (de Groot et al., 2009; Delmotte et al., 2009; Rubiano-Labrador et al., 2014). Collectively, these efforts promise an upcoming generation of tailored organisms with higher bio/phytoremediation efficiencies and lower costs (**Figure 3**).

In future, efforts should be made to develop strategies to improve the tolerance, uptake, and hyperaccumulation of heavy metals/metalloids using genomic and metabolic engineering approaches. Pathways that control the uptake, detoxification, transport from root to shoot tissues and translocation and hyperaccumulation in the aboveground storage tissues can be engineered using gene-stacking approaches (**Figure 3**). Additionally, genome editing strategies can be designed using TALENs (transcription activator like effectors nucleases) technology or the powerful CRISPR-Cas9 (clustered regularly interspaced short palindromic repeats) system to produce microbes/plants for bio/phytoremediation purposes (**Figure 3**). Recently, an efficient and successful CRISPR/Cas9-mediated targeted mutagenesis has been reported in *Populus* plants (Fan et al., 2013). This is a particularly interesting finding since *Populus* plants are known to be ideal plants for the phytoremediation of several toxic pollutants.

Additionally, efforts should be made to develop breeding programs to improve the biomass and growth habits of natural hyperaccumulators and breed those traits into non-food, high biomass, fast growing plants for commercial phytoremediation of heavy metals and metalloids. Furthermore, efforts should be made to combine the phytoremediation approach with bioenergy through the dual use of plants for phytoremediation and biofuel production on contaminated lands. This approach would be useful to phytoremediate contaminated sites and simultaneously produce renewable energy that can offset the costs of applying these type of methodologies.

## Author Contributions

All authors listed, have made substantial, direct and intellectual contribution to the work, and approved it for publication.

## Conflict of Interest Statement

The authors declare that the research was conducted in the absence of any commercial or financial relationships that could be construed as a potential conflict of interest.

## References

[B1] Abou-Shanab R. A. I. Angle J. S. Chaney R. L. (2006). Bacterial inoculants affecting nickel uptake by *Alyssum murale* from low, moderate and high Ni soils. *Soil Biol. Biochem.* 38 2882–2889. 10.1016/j.soilbio.2006.04.045

[B2] Albright J. C. Goering A. W. Doroghazi J. R. Metcalf W. W. Kelleher N. L. (2014). Strain-specific proteogenomics accelerates the discovery of natural products via their biosynthetic pathways. *J. Ind. Microbiol. Biotechnol.* 41 451–459. 10.1007/s10295-013-1373-4 24242000 PMC3946956

[B3] Almaguer-Cantú V. Morales-Ramos L. H. Balderas-Rentería I. (2011). Biosorption of lead (II) and cadmium (II) using *Escherichia coli* genetically engineered with mice metallothionein I. *Water Sci. Technol.* 63 1607–1613. 10.2166/wst.2011.225 21866758

[B4] Almeida S. M. Umeo S. H. Marcante R. C. Yokota M. E. Valle J. S. Dragunski D. C. (2015). Iron bioaccumulation in mycelium of *Pleurotus ostreatus*. *Braz. J. Microbiol.* 46 195–200. 10.1590/S1517-838246120130695 26221108 PMC4512064

[B5] Ashauer R. Hintermeister A. O’Connor I. Elumelu M. Hollender J. Escher B. I. (2012). Significance of xenobiotic metabolism for bioaccumulation kinetics of organic chemicals in *Gammarus pulex*. *Environ. Sci. Technol.* 46 3498–3508. 10.1021/es204611h 22321051 PMC3308200

[B6] Bae W. Mehra R. K. Mulchandani A. Chen W. (2001). Genetic engineering of *Escherichia coli* for enhanced uptake and bioaccumulation of mercury. *Appl. Environ. Microbiol.* 67 5335–5338. 10.1128/AEM.67.11.5335-5338.2001 11679366 PMC93311

[B7] Banik S. Das K. Islam M. Salimullah M. (2014). Recent advancements and challenges in microbial bioremediation of heavy metals contamination. *JSM Biotechnol. Biomed. Eng.* 2 1035.

[B8] Bao Z. Cobb R. E. Zhao H. (2016). Accelerated genome engineering through multiplexing. *Wiley Interdiscip. Rev. Syst. Biol. Med.* 8 5–21. 10.1002/wsbm.1319 26394307 PMC4715650

[B9] Beveridge T. J. (1989). Role of cellular design in bacterial metal accumulation and mineralization. *Annu. Rev. Microbiol.* 43 147–171. 10.1146/annurev.mi.43.100189.001051 2679352

[B10] Bienert G. P. Thorsen M. Schüssler M. D. Nilsson H. R. Wagner A. Tamás M. J. (2008). A subgroup of plant aquaporins facilitate the bi-directional diffusion of As(OH)3 and Sb(OH)3 across membranes. *BMC Biol.* 6:26 10.1186/1741-7007-6-26 PMC2442057 18544156

[B11] Bizily S. P. Rugh C. L. Meagher R. B. (2000). Phytodetoxification of hazardous organomercurials by genetically engineered plants. *Nat. Biotechnol.* 18 213–217. 10.1038/72678 10657131

[B12] Bizily S. P. Rugh C. L. Summers A. O. Meagher R. B. (1999). Phytoremediation of methylmercury pollution: merB expression in *Arabidopsis thaliana* confers resistance to organomercurials. *Proc. Natl. Acad. Sci. U.S.A.* 96 6808–6813. 10.1073/pnas.96.12.6808 10359794 PMC21997

[B13] Brar S. K. Verma M. Surampalli R. Y. Misra K. Tyagi R. D. Meunier N. (2006). Bioremediation of hazardous wastes—a review. *Pract. Period. Hazard. Toxic. Radioact. Waste Manag.* 10 59–72. 10.1061/(ASCE)1090-025X(2006)10:2(59)

[B14] Cakir O. Candar-Cakir B. Zhang B. (2016). Small RNA and degradome sequencing reveals important microRNA function in *Astragalus chrysochlorus* response to selenium stimuli. *Plant Biotechnol. J.* 14 543–556. 10.1111/pbi.12397 25998129 PMC11388920

[B15] Çakir Ö. Turgut-Kara N. Ari Ş. Zhang B. (2015). *De Novo* transcriptome assembly and comparative analysis elucidate complicated mechanism regulating *Astragalus chrysochlorus* response to selenium stimuli. *PLoS ONE* 10:e0135677 10.1371/journal.pone.0135677 PMC4592229 26431547

[B16] Carlot M. Giacomini A. Casella S. (2002). Aspects of plant-microbe interactions in heavy metal polluted soil. *Acta Biotechnol.* 22 13–20. 10.1002/1521-3846(200205)22:1/2<13::AID-ABIO13>3.0.CO;2-9

[B17] Catarecha P. Segura M. D. Franco-Zorrilla J. M. García-Ponce B. Lanza M. Solano R. (2007). A mutant of the *Arabidopsis* phosphate transporter PHT1;1 displays enhanced arsenic accumulation. *Plant Cell* 19 1123–1133. 10.1105/tpc.106.041871 17400898 PMC1867351

[B18] Che D. Meagher R. B. Heaton A. C. P. Lima A. Rugh C. L. Merkle S. A. (2003). Expression of mercuric ion reductase in Eastern cottonwood (*Populus deltoides*) confers mercuric ion reduction and resistance. *Plant Biotechnol. J.* 1 311–319. 10.1046/j.1467-7652.2003.00031.x 17163907

[B19] Chen Y. Unger M. Ntai I. McClure R. A. Albright J. C. Thomson R. J. (2013). Gobichelin A and B: mixed-ligand siderophores discovered using proteomics. *Medchemcomm* 4 233–238. 10.1039/C2MD20232H 23336063 PMC3547389

[B20] Choi D. H. Kwon Y. M. Kwon K. K. Kim S.-J. (2015). Complete genome sequence of *Novosphingobium pentaromativorans* US6-1(T). *Stand. Genomic Sci.* 10 107 10.1186/s40793-015-0102-1 PMC4653889 26594308

[B21] Chojnacka K. (2010). Biosorption and bioaccumulation–the prospects for practical applications. *Environ. Int.* 36 299–307. 10.1016/j.envint.2009.12.001 20051290

[B22] Chu B. C. Garcia-Herrero A. Johanson T. H. Krewulak K. D. Lau C. K. Peacock R. S. (2010). Siderophore uptake in bacteria and the battle for iron with the host; a bird’s eye view. *Biometals* 23 601–611. 10.1007/s10534-010-9361-x 20596754

[B23] Ciesielska K. Van Bogaert I. N. Chevineau S. Li B. Groeneboer S. Soetaert W. (2014). Exoproteome analysis of *Starmerella bombicola* results in the discovery of an esterase required for lactonization of sophorolipids. *J. Proteomics* 98 159–174. 10.1016/j.jprot.2013.12.026 24418522

[B24] Cobbina S. J. Chen Y. Zhou Z. Wu X. Zhao T. Zhang Z. (2015). Toxicity assessment due to sub-chronic exposure to individual and mixtures of four toxic heavy metals. *J. Hazard. Mater.* 294 109–120. 10.1016/j.jhazmat.2015.03.057 25863025

[B25] Cvjetko P. Zovko M. Balen B. (2014). Proteomics of heavy metal toxicity in plants. *Arh. Hig. Rada Toksikol.* 65 1–18. 10.2478/10004-1254-65-2014-2443 24526604

[B26] de Groot A. Dulermo R. Ortet P. Blanchard L. Guérin P. Fernandez B. (2009). Alliance of proteomics and genomics to unravel the specificities of Sahara bacterium *Deinococcus deserti*. *PLoS Genet.* 5:e1000434 10.1371/journal.pgen.1000434 PMC2669436 19370165

[B27] Delmotte N. Knief C. Chaffron S. Innerebner G. Roschitzki B. Schlapbach R. (2009). Community proteogenomics reveals insights into the physiology of phyllosphere bacteria. *Proc. Natl. Acad. Sci. U.S.A.* 106 16428–16433. 10.1073/pnas.0905240106 19805315 PMC2738620

[B28] Desai J. D. Banat I. M. (1997). Microbial production of surfactants and their commercial potential. *Microbiol. Mol. Biol. Rev.* 61 47–64. 9106364 10.1128/mmbr.61.1.47-64.1997 PMC232600

[B29] Dhankher O. P. Li Y. Rosen B. P. Shi J. Salt D. Senecoff J. F. (2002). Engineering tolerance and hyperaccumulation of arsenic in plants by combining arsenate reductase and gamma-glutamylcysteine synthetase expression. *Nat. Biotechnol.* 20 1140–1145. 10.1038/nbt747 12368812

[B30] Dhankher O. P. Pilon-Smits E. A. H. Meagher R. B. Doty S. (2011). “Biotechnological approaches for phytoremediation,” in *Plant Biotechnology and Agriculture*, eds Altman A. Hasegawa P. M. (Oxford: Academic Press), 309–328.

[B31] Diepens N. J. Dimitrov M. R. Koelmans A. A. Smidt H. (2015). Molecular assessment of bacterial community dynamics and functional end points during sediment bioaccumulation tests. *Environ. Sci. Technol.* 49 13586–13595. 10.1021/acs.est.5b02992 26466173

[B32] Dimkpa C. O. Svatos A. Dabrowska P. Schmidt A. Boland W. Kothe E. (2008). Involvement of siderophores in the reduction of metal-induced inhibition of auxin synthesis in *Streptomyces* spp. *Chemosphere* 74 19–25. 10.1016/j.chemosphere.2008.09.079 18986679

[B33] Ding J. Lu G. Li Y. (2016). Interactive effects of selected pharmaceutical mixtures on bioaccumulation and biochemical status in crucian carp (*Carassius auratus*). *Chemosphere* 148 21–31. 10.1016/j.chemosphere.2016.01.017 26800487

[B34] Diop M. Howsam M. Diop C. Goossens J. F. Diouf A. Amara R. (2016). Assessment of trace element contamination and bioaccumulation in algae (*Ulva lactuca*), mussels (*Perna perna*), shrimp (*Penaeus kerathurus*), and fish (*Mugil cephalus*, *Sarotherondon melanotheron*) along the Senegalese coast. *Mar. Pollut. Bull.* 103 339–343. 10.1016/j.marpolbul.2015.12.038 26763317

[B35] Dixit R. Wasiullah E. Malaviya D. Pandiyan K. Singh U. Sahu A. (2015). Bioremediation of heavy metals from soil and aquatic environment: an overview of principles and criteria of fundamental processes. *Sustainability* 7 2189–2212. 10.3390/su7022189

[B36] Dubey S. Shri M. Misra P. Lakhwani D. Bag S. K. Asif M. H. (2014). Heavy metals induce oxidative stress and genome-wide modulation in transcriptome of rice root. *Funct. Integr. Genomics* 14 401–417. 10.1007/s10142-014-0361-8 24553786

[B37] Dussa F. Mozafara A. Oertlia J. J. Jaeggib W. (1986). Effect of bacteria on the iron uptake by axenically-cultured roots of Fe-efficient and Fe-inefficient tomatoes (*Lycopersicon esculentum* Mill.). *J. Plant Nutr.* 9 587–598. 10.1080/01904168609363468

[B38] El-Metwally S. Ouda O. M. Helmy M. (2014). *Next Generation Sequencing Technologies and Challenges in Sequence Assembly*, 1st Edn. New York, NY: Springer, 10.1007/978-1-4939-0715-1

[B39] Evans K. M. Gatehouse J. A. Lindsay W. P. Shi J. Tommey A. M. Robinson N. J. (1992). Expression of the pea metallothionein-like gene PsMTA in *Escherichia coli* and *Arabidopsis thaliana* and analysis of trace metal ion accumulation: implications for PsMTA function. *Plant Mol. Biol.* 20 1019–1028. 10.1007/BF00028889 1463837

[B40] Fan X.-D. Wang J.-Q. Yang N. Dong Y.-Y. Liu L. Wang F.-W. (2013). Gene expression profiling of soybean leaves and roots under salt, saline-alkali and drought stress by high-throughput Illumina sequencing. *Gene* 512 392–402. 10.1016/j.gene.2012.09.100 23063936

[B41] Flathman P. E. Lanza G. R. (2010). Phytoremediation: current views on an emerging green technology. *J. Soil Contam.* 7 415–432.

[B42] Flissi A. Dufresne Y. Michalik J. Tonon L. Janot S. Noé L. (2016). Norine, the knowledgebase dedicated to non-ribosomal peptides, is now open to crowdsourcing. *Nucleic Acids Res.* 44 D1113–D1118. 10.1093/nar/gkv1143 26527733 PMC4702827

[B43] Fomina M. Gadd G. M. (2014). Biosorption: current perspectives on concept, definition and application. *Bioresour. Technol.* 160 3–14. 10.1016/j.biortech.2013.12.102 24468322

[B44] Galal T. M. Farahat E. A. (2015). The invasive macrophyte *Pistia stratiotes* L. as a bioindicator for water pollution in Lake Mariut, Egypt. *Environ. Monit. Assess.* 187 701 10.1007/s10661-015-4941-4 26497561

[B45] Gasic K. Korban S. S. (2007). Transgenic Indian mustard (*Brassica juncea*) plants expressing an *Arabidopsis* phytochelatin synthase (AtPCS1) exhibit enhanced As and Cd tolerance. *Plant Mol. Biol.* 64 361–369. 10.1007/s11103-007-9158-7 17390107

[B46] Gisbert C. Ros R. De Haro A. Walker D. J. Pilar Bernal M. Serrano R. (2003). A plant genetically modified that accumulates Pb is especially promising for phytoremediation. *Biochem. Biophys. Res. Commun.* 303 440–445. 10.1016/S0006-291X(03)00349-8 12659836

[B47] Glick B. R. (2003). Phytoremediation: synergistic use of plants and bacteria to clean up the environment. *Biotechnol. Adv.* 21 383–393. 10.1016/S0734-9750(03)00055-7 14499121

[B48] Gorfer M. Persak H. Berger H. Brynda S. Bandian D. Strauss J. (2009). Identification of heavy metal regulated genes from the root associated ascomycete *Cadophora finlandica* using a genomic microarray. *Mycol. Res.* 113 1377–1388. 10.1016/j.mycres.2009.09.005 19770041

[B49] He M. Li X. Liu H. Miller S. J. Wang G. Rensing C. (2011). Characterization and genomic analysis of a highly chromate resistant and reducing bacterial strain *Lysinibacillus fusiformis* ZC1. *J. Hazard. Mater.* 185 682–688. 10.1016/j.jhazmat.2010.09.072 20952126

[B50] He Z. Gentry T. J. Schadt C. W. Wu L. Liebich J. Chong S. C. (2007). GeoChip: a comprehensive microarray for investigating biogeochemical, ecological and environmental processes. *ISME J.* 1 67–77. 10.1038/ismej.2007.2 18043615

[B51] Heaton A. C. P. Rugh C. L. Kim T. Wang N. J. Meagher R. B. (2003). Toward detoxifying mercury-polluted aquatic sediments with rice genetically engineered for mercury resistance. *Environ. Toxicol. Chem.* 22 2940–2947. 10.1897/02-442 14713034

[B52] Heaton A. C. P. Rugh C. L. Wang N.-J. Meagher R. B. (2005). Physiological responses of transgenic merA-TOBACCO (*Nicotiana tabacum*) to foliar and root mercury exposure. *Water Air Soil Pollut.* 161 137–155. 10.1007/s11270-005-7111-4

[B53] Helmy M. Tomita M. Ishihama Y. (2012). Peptide identification by searching large-scale tandem mass spectra against large databases: bioinformatics methods in proteogenomics. *Gene Genome Genomics* 6 76–85.

[B54] Hiraoka S. Machiyama A. Ijichi M. Inoue K. Oshima K. Hattori M. (2016). Genomic and metagenomic analysis of microbes in a soil environment affected by the 2011 Great East Japan Earthquake tsunami. *BMC Genomics* 17:53 10.1186/s12864-016-2380-4 PMC4712596 26764021

[B55] Hirschi K. D. Korenkov V. D. Wilganowski N. L. Wagner G. J. (2000). Expression of *Arabidopsis* CAX2 in tobacco. Altered metal accumulation and increased manganese tolerance. *Plant Physiol.* 124 125–133. 10.1104/pp.124.1.125 10982428 PMC59128

[B56] Hossain M. A. Piyatida P. da Silva J. A. T. Fujita M. (2012). Molecular mechanism of heavy metal toxicity and tolerance in plants: central role of glutathione in detoxification of reactive oxygen species and methylglyoxal and in heavy metal chelation. *J. Bot.* 2012 872875 10.1155/2012/872875

[B57] Hossain Z. Komatsu S. (2012). Contribution of proteomic studies towards understanding plant heavy metal stress response. *Front. Plant Sci.* 3:310 10.3389/fpls.2012.00310 PMC3555118 23355841

[B58] Houston M. C. (2007). The role of mercury and cadmium heavy metals in vascular disease, hypertension, coronary heart disease, and myocardial infarction. *Altern. Ther. Health Med.* 13 S128–S133. 17405690

[B59] Hu P. Brodie E. L. Suzuki Y. McAdams H. H. Andersen G. L. (2005). Whole-genome transcriptional analysis of heavy metal stresses in *Caulobacter crescentus*. *J. Bacteriol.* 187 8437–8449. 10.1128/JB.187.24.8437-8449.2005 16321948 PMC1317002

[B60] IARC Monographs (2004). *IARC Monographs on the Evaluation of Carcinogenic Risks to Humans.* Available at: http://monographs.iarc.fr/ENG/Monographs/vol83/mono83.pdf

[B61] Italiano F. Buccolieri A. Giotta L. Agostiano A. Valli L. Milano F. (2009). Response of the carotenoidless mutant *Rhodobacter sphaeroides* growing cells to cobalt and nickel exposure. *Int. Biodeterior. Biodegradation* 63 948–957. 10.1016/j.ibiod.2009.05.001

[B62] Jackson S. A. Borchert E. O’Gara F. Dobson A. D. W. (2015). Metagenomics for the discovery of novel biosurfactants of environmental interest from marine ecosystems. *Curr. Opin. Biotechnol.* 33 176–182. 10.1016/j.copbio.2015.03.004 25812477

[B63] Jaiswar S. Kazi M. A. Mehta S. (2015). Bioaccumulation of heavy metals by freshwater algal species of Bhavnagar, Gujarat, India. *J. Environ. Biol.* 36 1361–1366. 26688974

[B64] Javanbakht V. Alavi S. A. Zilouei H. (2014). Mechanisms of heavy metal removal using microorganisms as biosorbent. *Water Sci. Technol.* 69 1775–1787. 10.2166/wst.2013.718 24804650

[B65] Jones O. A. H. Dias D. A. Callahan D. L. Kouremenos K. A. Beale D. J. Roessner U. (2015). The use of metabolomics in the study of metals in biological systems. *Metallomics* 7 29–38. 10.1039/c4mt00123k 25047028

[B66] Joutey N. T. Sayel H. Bahafid W. El Ghachtouli N. (2015). Mechanisms of hexavalent chromium resistance and removal by microorganisms. *Rev. Environ. Contam. Toxicol.* 233 45–69. 10.1007/978-3-319-10479-9_2 25367133

[B67] Khan M. U. Sessitsch A. Harris M. Fatima K. Imran A. Arslan M. (2014). Cr-resistant rhizo- and endophytic bacteria associated with *Prosopis juliflora* and their potential as phytoremediation enhancing agents in metal-degraded soils. *Front. Plant Sci.* 5:755 10.3389/fpls.2014.00755 PMC4284999 25610444

[B68] Kiran G. S. Ninawe A. S. Lipton A. N. Pandian V. Selvin J. (2015). Rhamnolipid biosurfactants: evolutionary implications, applications and future prospects from untapped marine resource. *Crit. Rev. Biotechnol.* 2 1–17. 10.3109/07388551.2014.979758 25641324

[B69] Koedam N. Wittouck E. Gaballa A. Gillis A. Hofte M. Cornelis P. (1994). Detection and differentiation of microbial siderophores by isoelectric focusing and chrome azurol S overlay. *Biometals* 7 287–291. 10.1007/BF00144123 7812113

[B70] Kotrba P. Dolecková L. de Lorenzo V. Ruml T. (1999). Enhanced bioaccumulation of heavy metal ions by bacterial cells due to surface display of short metal binding peptides. *Appl. Environ. Microbiol.* 65 1092–1098. 10049868 10.1128/aem.65.3.1092-1098.1999 PMC91149

[B71] Kotrba P. Najmanova J. Macek T. Ruml T. Mackova M. (2009). Genetically modified plants in phytoremediation of heavy metal and metalloid soil and sediment pollution. *Biotechnol. Adv.* 27 799–810. 10.1016/j.biotechadv.2009.06.003 19567265

[B72] Krishnaswamy R. Wilson D. B. (2000). Construction and characterization of an *Escherichia coli* strain genetically engineered for Ni(II) bioaccumulation. *Appl. Environ. Microbiol.* 66 5383–5386. 10.1128/AEM.66.12.5383-5386.2000 11097917 PMC92471

[B73] Kumar A. Kage U. Mosa K. Dhokane D. (2014). Metabolomics: a novel tool to bridge phenome to genome under changing climate to ensure food security. *Med. Aromat. Plants* 3:e154 10.4172/2167-0412.1000e154

[B74] Kumar K. Mosa K. A. Chhikara S. Musante C. White J. C. Dhankher O. P. (2014). Two rice plasma membrane intrinsic proteins, OsPIP2;4 and OsPIP2;7, are involved in transport and providing tolerance to boron toxicity. *Planta* 239 187–198. 10.1007/s00425-013-1969-y 24142111

[B75] Lampis S. Santi C. Ciurli A. Andreolli M. Vallini G. (2015). Promotion of arsenic phytoextraction efficiency in the fern *Pteris vittata* by the inoculation of As-resistant bacteria: a soil bioremediation perspective. *Front. Plant Sci.* 6:80 10.3389/fpls.2015.00080 PMC4332284 25741356

[B76] LeDuc D. L. Tarun A. S. Montes-Bayon M. Meija J. Malit M. F. Wu C. P. (2004). Overexpression of selenocysteine methyltransferase in *Arabidopsis* and Indian mustard increases selenium tolerance and accumulation. *Plant Physiol.* 135 377–383. 10.1104/pp.103.026989 14671009 PMC429391

[B77] Leung P. T. Y. Ip J. C. H. Mak S. S. T. Qiu J. W. Lam P. K. S. Wong C. K. C. (2014). De novo transcriptome analysis of *Perna viridis* highlights tissue-specific patterns for environmental studies. *BMC Genomics* 15:804 10.1186/1471-2164-15-804 PMC4190305 25239240

[B78] Li Q. Yu Z. Shao X. He J. Li L. (2009). Improved phosphate biosorption by bacterial surface display of phosphate-binding protein utilizing ice nucleation protein. *FEMS Microbiol. Lett.* 299 44–52. 10.1111/j.1574-6968.2009.01724.x 19686343

[B79] Li Y. Dhankher O. P. Carreira L. Lee D. Chen A. Schroeder J. I. (2004). Overexpression of phytochelatin synthase in *Arabidopsis* leads to enhanced arsenic tolerance and cadmium hypersensitivity. *Plant Cell Physiol.* 45 1787–1797. 10.1093/pcp/pch202 15653797

[B80] Liang Zhu Y. Pilon-Smits E. Jouanin L. Terry N. (1999). Overexpression of glutathione synthetase in indian mustard enhances cadmium accumulation and tolerance. *Plant Physiol.* 119 73–80. 10.1104/pp.119.1.73 9880348 PMC32244

[B81] Ma Y. Oliveira R. S. Wu L. Luo Y. Rajkumar M. Rocha I. (2015). Inoculation with metal-mobilizing plant-growth-promoting rhizobacterium *Bacillus* sp. SC2b and its role in rhizoremediation. *J. Toxicol. Environ. Health A* 78 931–944. 10.1080/15287394.2015.1051205 26167758

[B82] Maier R. M. Pepper I. L. Gerba C. P. (2000). *Environmental Microbiology.* Houston, TX: Gulf Professional Publishing.

[B83] Maier R. M. Soberón-Chávez G. (2000). *Pseudomonas aeruginosa* rhamnolipids: biosynthesis and potential applications. *Appl. Microbiol. Biotechnol.* 54 625–633. 10.1007/s002530000443 11131386

[B84] Matés J. M. Segura J. A. Alonso F. J. Márquez J. (2010). Roles of dioxins and heavy metals in cancer and neurological diseases using ROS-mediated mechanisms. *Free Radic. Biol. Med.* 49 1328–1341. 10.1016/j.freeradbiomed.2010.07.028 20696237

[B85] Meagher R. B. (2000). Phytoremediation of toxic elemental and organic pollutants. *Curr. Opin. Plant Biol.* 3 153–162. 10.1016/S1369-5266(00)00108-4 10712958

[B86] Mishra A. Malik A. (2013). Recent advances in microbial metal bioaccumulation. *Crit. Rev. Environ. Sci. Technol.* 43 1162–1222. 10.1080/10934529.2011.627044

[B87] Mnif I. Ghribi D. (2015). Review lipopeptides biosurfactants: mean classes and new insights for industrial, biomedical, and environmental applications. *Biopolymers* 104 129–147. 10.1002/bip.22630 25808118

[B88] Mok J. S. Yoo H. D. Kim P. H. Yoon H. D. Park Y. C. Lee T. S. (2015). Bioaccumulation of heavy metals in oysters from the southern coast of Korea: assessment of potential risk to human health. *Bull. Environ. Contam. Toxicol.* 94 749–755. 10.1007/s00128-015-1534-4 25863478

[B89] Moreno F. N. Anderson C. W. N. Stewart R. B. Robinson B. H. (2005). Mercury volatilisation and phytoextraction from base-metal mine tailings. *Environ. Pollut.* 136 341–352. 10.1016/j.envpol.2004.11.020 15840542

[B90] Mosa K. A. (2012). *Functional Characterization of Members of Plasma Membrane Intrinsic Proteins Subfamily and their Involvement in Metalloids Transport in Plants.* Doctoral dissertations, University of Massachusetts Amherst, Amherst, MA, 1–134.

[B91] Mosa K. A. Kumar K. Chhikara S. Dhankher O. P. (2011). “The role of rice plasma membrane intrinsic proteins in boron transport,” in *Proceedings of the In Vitro Cellular & Developmental Biology-Animal*, Vol. 47 (New York, NY: Springer), S77.

[B92] Mosa K. A. Kumar K. Chhikara S. Mcdermott J. Liu Z. Musante C. (2012). Members of rice plasma membrane intrinsic proteins subfamily are involved in arsenite permeability and tolerance in plants. *Transgenic Res.* 21 1265–1277. 10.1007/s11248-012-9600-8 22350764

[B93] Mosa K. A. Kumar K. Chhikara S. Musante C. White J. C. Dhankher O. P. (2016). Enhanced boron tolerance in plants mediated by bidirectional transport through plasma membrane intrinsic proteins. *Sci. Rep.* 6:21640 10.1038/srep21640 PMC4763227 26902738

[B94] Müller M. M. Kügler J. H. Henkel M. Gerlitzki M. Hörmann B. Pöhnlein M. (2012). Rhamnolipids–next generation surfactants? *J. Biotechnol.* 162 366–380. 10.1016/j.jbiotec.2012.05.022 22728388

[B95] Mulligan C. N. (2005). Environmental applications for biosurfactants. *Environ. Pollut.* 133 183–198. 10.1016/j.envpol.2004.06.009 15519450

[B96] Muralidharan S. Thompson E. Raftos D. Birch G. Haynes P. A. (2012). Quantitative proteomics of heavy metal stress responses in Sydney rock oysters. *Proteomics* 12 906–921. 10.1002/pmic.201100417 22539440

[B97] Naz T. Khan M. D. Ahmed I. Rehman S. U. Rha E. S. Malook I. (2015). Biosorption of heavy metals by *Pseudomonas* species isolated from sugar industry. *Toxicol. Ind. Health* 10.1177/0748233715569900 [Epub ahead of print]. 25739395

[B98] Neilands J. B. (1995). Siderophores: structure and function of microbial iron transport compounds. *J. Biol. Chem.* 270 26723–26726. 10.1074/jbc.270.45.26723 7592901

[B99] Neubauer U. Furrer G. Kayser A. Schulin R. (2000). Siderophores, NTA, and citrate: potential soil amendments to enhance heavy metal mobility in phytoremediation. *Int. J. Phytoremediation* 2 353–368. 10.1080/15226510008500044

[B100] Nilanjana D. Vimala R. Karthika K. (2008). Biosorption of heavy metals–An overview. *Indian J. Biotechnol.* 7 159–169.

[B101] Ochsner U. A. Fiechter A. Reiser J. (1994). Isolation, characterization, and expression in *Escherichia coli* of the *Pseudomonas aeruginosa* rhlAB genes encoding a rhamnosyltransferase involved in rhamnolipid biosurfactant synthesis. *J. Biol. Chem.* 269 19787–19795. 8051059

[B102] Oh K. Li T. Cheng H. Hu X. He C. Yan L. (2013). Development of profitable phytoremediation of contaminated soils with biofuel crops. *J. Environ. Prot.* 4 58–64. 10.4236/jep.2013.44A008

[B103] Oliveira J. S. Araújo W. Lopes Sales A. I. de Brito Guerra A. da Silva Araújo S. C. de Vasconcelos A. T. R. (2015). BioSurfDB: knowledge and algorithms to support biosurfactants and biodegradation studies. *Database* 2015 bav033. 10.1093/database/bav033 PMC4381105 25833955

[B104] Ovečka M. Takáč T. (2014). Managing heavy metal toxicity stress in plants: biological and biotechnological tools. *Biotechnol. Adv.* 32 73–86. 10.1016/j.biotechadv.2013.11.011 24333465

[B105] Park H.-M. Kim B.-G. Chang D. Malla S. Joo H.-S. Kim E.-J. (2013). Genome-based cryptic gene discovery and functional identification of NRPS siderophore peptide in *Streptomyces peucetius*. *Appl. Microbiol. Biotechnol.* 97 1213–1222. 10.1007/s00253-012-4268-9 22825833

[B106] Pazirandeh M. Wells B. M. Ryan R. L. (1998). Development of bacterium-based heavy metal biosorbents: enhanced uptake of cadmium and mercury by *Escherichia coli* expressing a metal binding motif. *Appl. Environ. Microbiol.* 64 4068–4072. 9758845 10.1128/aem.64.10.4068-4072.1998 PMC106604

[B107] Peña-Montenegro T. D. Dussán J. (2013). Genome sequence and description of the heavy metal tolerant bacterium *Lysinibacillus sphaericus* strain OT4b.31. *Stand. Genomic Sci.* 9 42–56. 10.4056/sigs.4227894 24501644 PMC3910547

[B108] Pidlisnyuk V. Stefanovska T. Lewis E. E. Erickson L. E. Davis L. C. (2014). Miscanthus as a productive biofuel crop for phytoremediation. *Crit. Rev. Plant Sci.* 33 1–19. 10.1080/07352689.2014.847616

[B109] Pilon M. Owen J. D. Garifullina G. F. Kurihara T. Mihara H. Esaki N. (2003). Enhanced selenium tolerance and accumulation in transgenic *Arabidopsis* expressing a mouse selenocysteine lyase. *Plant Physiol.* 131 1250–1257. 10.1104/pp.102.014639 12644675 PMC166885

[B110] Pilon-Smits E. (2005). Phytoremediation. *Annu. Rev. Plant Biol.* 56 15–39. 10.1146/annurev.arplant.56.032604.144214 15862088

[B111] Pilon-Smits E. Hwang S. Mel Lytle C. Zhu Y. Tai J. Bravo R. (1999). Overexpression of ATP sulfurylase in indian mustard leads to increased selenate uptake, reduction, and tolerance. *Plant Physiol.* 119 123–132. 10.1104/pp.119.1.123 9880353 PMC32211

[B112] Pilon-Smits E. A. H. LeDuc D. L. (2009). Phytoremediation of selenium using transgenic plants. *Curr. Opin. Biotechnol.* 20 207–212. 10.1016/j.copbio.2009.02.001 19269806

[B113] Rajkumar M. Ae N. Prasad M. N. V. Freitas H. (2010). Potential of siderophore-producing bacteria for improving heavy metal phytoextraction. *Trends Biotechnol.* 28 142–149. 10.1016/j.tibtech.2009.12.002 20044160

[B114] Reznik G. O. Vishwanath P. Pynn M. A. Sitnik J. M. Todd J. J. Wu J. (2010). Use of sustainable chemistry to produce an acyl amino acid surfactant. *Appl. Microbiol. Biotechnol.* 86 1387–1397. 10.1007/s00253-009-2431-8 20094712

[B115] Romera E. González F. Ballester A. Blázquez M. L. Muñoz J. A. (2006). Biosorption with algae: a statistical review. *Crit. Rev. Biotechnol.* 26 223–235. 10.1080/07388550600972153 17095433

[B116] Ron E. Z. Rosenberg E. (2001). Natural roles of biosurfactants. *Environ. Microbiol.* 3 229–236. 10.1046/j.1462-2920.2001.00190.x 11359508

[B117] Rubiano-Labrador C. Bland C. Miotello G. Guérin P. Pible O. Baena S. (2014). Proteogenomic insights into salt tolerance by a halotolerant alpha-proteobacterium isolated from an Andean saline spring. *J. Proteomics* 97 36–47. 10.1016/j.jprot.2013.05.020 23727365

[B118] Rufino R. D. Luna J. M. Campos-Takaki G. M. Ferreira S. R. M. Sarubbo L. A. (2012). Application of the biosurfactant produced by *Candida lipolytica* in the remediation of heavy metals. *Chem. Eng. Trans.* 27 61–66.

[B119] Rugh C. L. Senecoff J. F. Meagher R. B. Merkle S. A. (1998). Development of transgenic yellow poplar for mercury phytoremediation. *Nat. Biotechnol.* 16 925–928. 10.1038/nbt1098-925 9788347

[B120] Rugh C. L. Wilde H. D. Stack N. M. Thompson D. M. Summers A. O. Meagher R. B. (1996). Mercuric ion reduction and resistance in transgenic *Arabidopsis thaliana* plants expressing a modified bacterial merA gene. *Proc. Natl. Acad. Sci. U.S.A.* 93 3182–3187. 10.1073/pnas.93.8.3182 8622910 PMC39579

[B121] Ruiz O. N. Daniell H. (2009). Genetic engineering to enhance mercury phytoremediation. *Curr. Opin. Biotechnol.* 20 213–219. 10.1016/j.copbio.2009.02.010 19328673 PMC2692567

[B122] Salt D. E. Blaylock M. Kumar N. P. Dushenkov V. Ensley B. D. Chet I. (1995). Phytoremediation: a novel strategy for the removal of toxic metals from the environment using plants. *Nat. Biotechnol.* 13 468–474. 10.1038/nbt0595-468 9634787

[B123] Salt D. E. Smith R. D. Raskin I. (1998). PHYTOREMEDIATION. *Annu. Rev. Plant Physiol. Plant Mol. Biol.* 49 643–668. 10.1146/annurev.arplant.49.1.643 15012249

[B124] Samuelson P. Wernérus H. Svedberg M. Ståhl S. (2000). Staphylococcal surface display of metal-binding polyhistidyl peptides. *Appl. Environ. Microbiol.* 66 1243–1248. 10.1128/AEM.66.3.1243-1248.2000 10698802 PMC91973

[B125] Schalk I. J. Hannauer M. Braud A. (2011). New roles for bacterial siderophores in metal transport and tolerance. *Environ. Microbiol.* 13 2844–2854. 10.1111/j.1462-2920.2011.02556.x 21883800

[B126] Scragg A. (2005). *Environmental Biotechnology*, 2nd Edn. New York, NY: Oxford University Press.

[B127] Searle L. J. Méric G. Porcelli I. Sheppard S. K. Lucchini S. (2015). Variation in siderophore biosynthetic gene distribution and production across environmental and faecal populations of *Escherichia coli*. *PLoS ONE* 10:e0117906 10.1371/journal.pone.0117906 PMC4355413 25756870

[B128] Sharma A. Johri B. N. (2003). Growth promoting influence of siderophore-producing *Pseudomonas* strains GRP3A and PRS9 in maize (*Zea mays* L.) under iron limiting conditions. *Microbiol. Res.* 158 243–248. 10.1078/0944-5013-00197 14521234

[B129] Shi B. Huang Z. Xiang X. Huang M. Wang W.-X. Ke C. (2015). Transcriptome analysis of the key role of GAT2 gene in the hyper-accumulation of copper in the oyster *Crassostrea angulata*. *Sci. Rep.* 5 17751 10.1038/srep17751 PMC4673431 26648252

[B130] Sinha S. Mukherjee S. K. (2008). Cadmium-induced siderophore production by a high Cd-resistant bacterial strain relieved Cd toxicity in plants through root colonization. *Curr. Microbiol.* 56 55–60. 10.1007/s00284-007-9038-z 17899260

[B131] Somdee T. Thathong B. Somdee A. (2015). The removal of cyanobacterial hepatotoxin [Dha(7)] microcystin-LR via bioaccumulation in water lettuce (*Pistia stratiotes* L.). *Bull. Environ. Contam. Toxicol.* 10.1007/s00128-015-1715-1 [Epub ahead of print]. 26687499

[B132] Song W.-Y. Sohn E. J. Martinoia E. Lee Y. J. Yang Y.-Y. Jasinski M. (2003). Engineering tolerance and accumulation of lead and cadmium in transgenic plants. *Nat. Biotechnol.* 21 914–919. 10.1038/nbt850 12872132

[B133] Stadnicka J. Schirmer K. Ashauer R. (2012). Predicting concentrations of organic chemicals in fish by using toxicokinetic models. *Environ. Sci. Technol.* 46 3273–3280. 10.1021/es2043728 22324398 PMC3308199

[B134] Stone E. L. Timmer V. R. (1975). On the copper content of some northern conifers. *Can. J. Bot.* 53 1453–1456. 10.1139/b75-177

[B135] Sunkar R. Kaplan B. Bouché N. Arazi T. Dolev D. Talke I. N. (2000). Expression of a truncated tobacco NtCBP4 channel in transgenic plants and disruption of the homologous *Arabidopsis* CNGC1 gene confer Pb2+ tolerance. *Plant J.* 24 533–542. 10.1046/j.1365-313x.2000.00901.x 11115134

[B136] Tan B. Ng C. Nshimyimana J. P. Loh L. L. Gin K. Y.-H. Thompson J. R. (2015). Next-generation sequencing (NGS) for assessment of microbial water quality: current progress, challenges, and future opportunities. *Front. Microbiol.* 6:1027 10.3389/fmicb.2015.01027 PMC4585245 26441948

[B137] Tran V. S. Ngo H. H. Guo W. Zhang J. Liang S. Ton-That C. (2015). Typical low cost biosorbents for adsorptive removal of specific organic pollutants from water. *Bioresour. Technol.* 182 353–363. 10.1016/j.biortech.2015.02.003 25690682

[B138] Van Nostrand J. D. Wu W.-M. Wu L. Deng Y. Carley J. Carroll S. (2009). GeoChip-based analysis of functional microbial communities during the reoxidation of a bioreduced uranium-contaminated aquifer. *Environ. Microbiol.* 11 2611–2626. 10.1111/j.1462-2920.2009.01986.x 19624708

[B139] Velásquez L. Dussan J. (2009). Biosorption and bioaccumulation of heavy metals on dead and living biomass of *Bacillus sphaericus*. *J. Hazard. Mater.* 167 713–716. 10.1016/j.jhazmat.2009.01.044 19201532

[B140] Vijayaraghavan K. Yun Y.-S. (2008). Bacterial biosorbents and biosorption. *Biotechnol. Adv.* 26 266–291. 10.1016/j.biotechadv.2008.02.002 18353595

[B141] Wang J. Chen C. (2008). Biosorbents for heavy metals removal and their future. *Biotechnol. Adv.* 27 195–226. 10.1016/j.biotechadv.2008.11.002 19103274

[B142] Wang Y. Dong C. Xue Z. Jin Q. Xu Y. (2016). De novo transcriptome sequencing and discovery of genes related to copper tolerance in *Paeonia ostii*. *Gene* 576 126–135. 10.1016/j.gene.2015.09.077 26435192

[B143] Wang Y. Zhao Z. Deng M. Liu R. Niu S. Fan G. (2015). Identification and functional analysis of microRNAs and their targets in *Platanus acerifolia* under lead (Pb) stress. *Int. J. Mol. Sci.* 16 7098–7111. 10.3390/ijms16047098 25830479 PMC4425006

[B144] Wei Y.-F. Li T. Li L.-F. Wang J.-L. Cao G.-H. Zhao Z.-W. (2016). Functional and transcript analysis of a novel metal transporter gene EpNramp from a dark septate endophyte (*Exophiala pisciphila*). *Ecotoxicol. Environ. Saf.* 124 363–368. 10.1016/j.ecoenv.2015.11.008 26595509

[B145] Wernérus H. Lehtiö J. Teeri T. Nygren P. A. Ståhl S. (2001). Generation of metal-binding staphylococci through surface display of combinatorially engineered cellulose-binding domains. *Appl. Environ. Microbiol.* 67 4678–4684. 10.1128/AEM.67.10.4678-4684.2001 11571172 PMC93219

[B146] Weyens N. van der Lelie D. Taghavi S. Vangronsveld J. (2009). Phytoremediation: plant-endophyte partnerships take the challenge. *Curr. Opin. Biotechnol.* 20 248–254. 10.1016/j.copbio.2009.02.012 19327979

[B147] Xie P. Hao X. Herzberg M. Luo Y. Nies D. H. Wei G. (2015). Genomic analyses of metal resistance genes in three plant growth promoting bacteria of legume plants in Northwest mine tailings, China. *J. Environ. Sci.* 27 179–187. 10.1016/j.jes.2014.07.017 25597676

[B148] Xie Y. Ye S. Wang Y. Xu L. Zhu X. Yang J. (2015). Transcriptome-based gene profiling provides novel insights into the characteristics of radish root response to Cr stress with next-generation sequencing. *Front. Plant Sci.* 6:202 10.3389/fpls.2015.00202 PMC4379753 25873924

[B149] Xiong J. Wu L. Tu S. Van Nostrand J. D. He Z. Zhou J. (2010). Microbial communities and functional genes associated with soil arsenic contamination and the rhizosphere of the arsenic-hyperaccumulating plant *Pteris vittata* L. *Appl. Environ. Microbiol.* 76 7277–7284. 10.1128/AEM.00500-10 20833780 PMC2976218

[B150] Xu L. Wang Y. Liu W. Wang J. Zhu X. Zhang K. (2015). De novo sequencing of root transcriptome reveals complex cadmium-responsive regulatory networks in radish (*Raphanus sativus* L.). *Plant Sci.* 236 313–323. 10.1016/j.plantsci.2015.04.015 26025544

[B151] Zakeri F. Sadeghizadeh M. Kardan M. R. Shahbani Zahiri H. Ahmadian G. Masoumi F. (2012). Differential proteome analysis of a selected bacterial strain isolated from a high background radiation area in response to radium stress. *J. Proteomics* 75 4820–4832. 10.1016/j.jprot.2012.05.020 22634040

[B152] Zeng H. Xu L. Singh A. Wang H. Du L. Poovaiah B. W. (2015). Involvement of calmodulin and calmodulin-like proteins in plant responses to abiotic stresses. *Front. Plant Sci.* 6:600 10.3389/fpls.2015.00600 PMC4532166 26322054

[B153] Zhao D. Li T. Shen M. Wang J. Zhao Z. (2015). Diverse strategies conferring extreme cadmium (Cd) tolerance in the dark septate endophyte (DSE), *Exophiala pisciphila*: evidence from RNA-seq data. *Microbiol. Res.* 170 27–35. 10.1016/j.micres.2014.09.005 25294257

[B154] Zhao F. J. Ma J. F. Meharg A. A. McGrath S. P. (2009). Arsenic uptake and metabolism in plants. *New Phytol.* 181 777–794. 10.1111/j.1469-8137.2008.02716.x 19207683

[B155] Zhou W. Zhang Y. Ding X. Liu Y. Shen F. Zhang X. (2012). Magnetotactic bacteria: promising biosorbents for heavy metals. *Appl. Microbiol. Biotechnol.* 95 1097–1104. 10.1007/s00253-012-4245-3 22763846

[B156] Zhu Y.-G. Rosen B. P. (2009). Perspectives for genetic engineering for the phytoremediation of arsenic-contaminated environments: from imagination to reality? *Curr. Opin. Biotechnol.* 20 220–224. 10.1016/j.copbio.2009.02.011 19303764 PMC4578631

[B157] Zwolak I. Zaporowska H. (2012). Selenium interactions and toxicity: a review. Selenium interactions and toxicity. *Cell Biol. Toxicol.* 28 31–46. 10.1007/s10565-011-9203-9 21913064

